# Bioengineering Microgels and Hydrogel Microparticles for Sensing Biomolecular Targets

**DOI:** 10.3390/gels3020020

**Published:** 2017-05-30

**Authors:** Edmondo Battista, Filippo Causa, Paolo Antonio Netti

**Affiliations:** 1 Interdisciplinary Research Centre on Biomaterials (CRIB) and Dipartimento di Ingegneria Chimica, dei Materiali e della Produzione Industriale (DICMAPI), University of Naples Federico II, Piazzale Tecchio 80, 80125 Napoli, Italy; edmondo.battista@unina.it (E.B.); nettipa@unina.it (P.A.N.); 2 Center for Advanced Biomaterials for HealthCare@CRIB, Istituto Italiano di Tecnologia, Largo Barsanti e Matteucci 53, 80125 Napoli, Italy

**Keywords:** microgels, hydrogel microparticles, multiplex bead-based assay, core/shell microgels, fluorescent enconding, oligonucleotide probes, immunoassays, microRNAs

## Abstract

Hydrogels, and in particular microgels, are playing an increasingly important role in a diverse range of applications due to their hydrophilic, biocompatible, and highly flexible chemical characteristics. On this basis, solution-like environment, non-fouling nature, easy probe accessibility and target diffusion, effective inclusion of reporting moieties can be achieved, making them ideal substrates for bio-sensing applications. In fact, hydrogels are already successfully used in immunoassays as well as sensitive nucleic acid assays, also enabling hydrogel-based suspension arrays. In this review, we discuss key parameters of hydrogels in the form of micron-sized particles to be used in sensing applications, paying attention to the protein and oligonucleotides (i.e., miRNAs) targets as most representative kind of biomarkers.

## 1. Introduction

Hydrogels microparticles and microgels are polymeric three-dimensional networks that largely swell in water without dissolving covering the nano- to micro-size range with superior features respect to the other conventional polymers. Recent research has revolved around such particular polymers as a highly versatile material platform to realize next generation of diagnostic tools [1,2,3]. “Bead-based multiplex assays” represent an evolution of traditional microarray platforms so that microparticles manufacturing is increasingly expanding. Multiplexed assays, where multiple tests or “panels” are performed on the same sample, offer the great advantage of carrying out in a single reaction vessel a number of analysis with volume equivalent to one well of a traditional enzyme-linked immunosorbent assay (ELISA) [4]. Although different types of microparticles are already on the market (e.g., fluorescent labeled and magnetic), there is a need for materials with high flexibility to introduce the right functionalities for the encoding and for the capture elements with enhanced properties.

In that context, microgels and hydrogel microparticles embody a chemical toolbox for the on-demand realization of polymer particles with different features addressing the needs of the final application by using combinatory approaches and microfluidic devices [5].

The most attractive characteristic of hydrogels relies on the flexible synthetic approaches to design multifunctional particles provided with suitable encoding and sensing capabilities toward individual targets such as pollutants [6] or clinically relevant biomarkers [7,8]. Multifunctional particles entail a differentiated control over the chemistry of the surface and the bulk to accommodate chemical compounds as elements to provide encoding and sensing molecules for the desired purpose.

Bead-based arrays have accounted for significant improvement respect to planar arrays for several synthetic and handling aspects. Regardless of the realization process (i.e., photolithography and robotic spotting) of planar arrays, the number of positions produced simultaneously is relatively limited and the capture molecules have to be conjugated one-by-one to every single position, while, in the case of microparticles, their attachment is realized at the same time on millions of beads with unmet reproducibility [9]. Indeed, the most problematic issue of the planar array relies on fact that all capture molecules have to be conjugate in the same conditions to the same surface chemistry. Microparticle technology allows producing separate batches where it is possible to attach different probe molecules at different ratios by using a different chemistry more appropriate for the chosen probe. 2D arrays are restricted to the predetermined set of panels, while, for microparticles, the panel test change is easily allowed thanks the versatility in the addition or subtraction of different population of captures molecules.

Furthermore, on planar arrays the hybridization and binding rates are slowed because of diffusion limitation at surface, whereas the efficient mixing realized whit microparticles accelerate the kinetics of recognition. Microparticles enable an easy handling with separation and washing steps realized in very smooth and fast fashion, and may even allow these to be eliminated altogether. The production costs of microparticles can be more and more reduced as the minute number used per reaction. Further, the interrogation of many particles per target molecule allows high statistical meaning of the data reflecting on the quality of entire assay.

High throughput microparticles analyses can be performed on miniaturized microfluidic systems matching the requirements of integrated low-cost devices for point-of-care clinical diagnostic applications. Microparticles have the potential to be sorted for a post analytical tasks into different wells or used in a combinatorial synthesis (i.e., split-and-mix approach) where capture probe can be progressively and combinatorially mounted on the beads [10].

**Figure 1:**
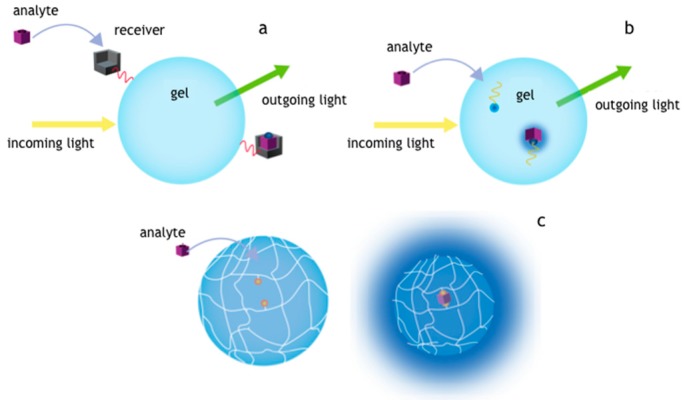
Schemes for proteins and oligonucleotides detection implemented in microgels and hydrogel microparticles with optical fluorescence read-out. In (**a**) is depicted the recognition of the target by a ligand placed on the surface of the gel, in (**b**) the capture molecules are copolymerized inside the hydrogel network. In (**c**) is reported a scheme of recognition of two distinct regions on the target and a transduction mechanism of the capture based on volume phase changes or Förster resonance energy transfer (FRET) [16].

In this review, we focus on applications of microgels and hydrogel microparticles in bioassay based on optical detection scheme for proteins or oligonucleotides (Figure 1) [11,12]. In particular, for proteins classical schemes of functionalization where antibodies are adapted in hydrogels, while most advanced system are based on peptides or aptamers copolymerized inside as capture agents. In the case of oligonucleotides, platforms reviewed here rely on sophisticated mechanisms allowing catching information on binding event in fluorescence [13]. Such probes can be implemented inside the hydrogels and are based on intelligent probes designed on specific base pairing [14,15].

Generally speaking, the first mechanism (Figure 1a) is based on a material provided with a molecular catcher placed on the surface that intercepts specifically the given target. Such mechanism is basically the one of the ELISAs and uses antibodies to capture small molecules or protein biomarkers. A secondary antibody fluorescently labeled can show the binding events the intrinsic fluorescence of captured molecule can be exploited simplifying the assay steps. This scheme can be also adapted to hydrogels even though diffusional barrier represents the most important issue for microparticle. Upon copolymerization with supportive monomers, capture elements can be incorporated in the inner part of the hydrogel network (Figure 1b). In that case, oligonucleotides or peptides can be covalently linked inside the matrix with a suitable porogen that guarantee good accessibility to the marker molecules. For this case the binding events can be shown in fluorescence by using an intelligent probe, as in the case of oligonucleotides, or exploiting the intrinsic fluorescence of target molecule. Another reported scheme (Figure 1c) provides volume changes of the hydrogel upon the recognition of given target in two distinct regions that may produce a collapse of the structure. The fluorescence optical detection in this case could be realized by Förster resonance energy transfer (FRET) system that come in contact after a specific binding event. In such scheme, the binding event can be also detected as a change of optical density or more complicated outputs involving differential diffraction of light or when coupled to nano- or microdevices (i.e., microcantilevers) can physically actuate a specific response [17,18].

## 2. Hydrogels: Macrogels, Microparticles and Microgels

Hydrogels, by definition, are three-dimensional cross-linked networks that can uptake and retain huge amount of water with respect to the mass of polymer [1,19,20]. Hydrogel can be primarily categorized for their dimensions and formats as either macrogels or microgels. Monolithic bulk networks are typically defined as macrogels ranging in size of hundreds of microns or greater. Hydrogels microparticles ranging from tens to hundreds of microns can also be considered a sort of macrogels. Microgels are colloidal water swellable networks with typical diameter ranges from 100 nm to 1 μm (sub-micrometer) [21,22]. Microgels are physically different from macrogels even though internally have the same gel structure. Indeed, as the high surface to volume ratios are several orders of magnitude larger than those in macrogels, microgels can present a non-uniform distribution of polymer chains throughout the network [23,24,25].

Further classifications of hydrogels can be done reflecting the structural composition and the response to the environment or specific stimuli. The way the cross-links are formed represents one of the first classifications of such materials between physical and chemical [26]. Physical gels are defined as entangled networks of polymer chains forming a three-dimensional network via non-covalent interactions. Such hydrogels can be reversibly dissolved in certain condition (i.e., pH, temperature, and ionic strength) able to weaken the hydrogen bonding, electrostatic or hydrophobic forces. Chemical cross-linking improves the gel stability, thanks to the formation of covalent bonds between the polymer chains, withstanding different environment conditions [27].

Another classification of hydrogel materials can be done according to the responsivity to the surrounding environment [28]. Polymeric networks that swell upon exposure to water can be defined as non-responsive, while responsive gels present additional feature displaying changes in solvation in response to certain stimuli such as temperature [29,30], pH [31], ionic strength [32], light [33], and electric field [34]. As result, this behavior accounts for different applications in many fields including biotechnology and biomedicine [35], where the reversible volume changes (sometimes as large as several hundred times the original volume) have been reported in response to minute changes in external environmental conditions. Here, we report on the synthesis, chemical modification and physical properties of common microgels and hydrogel microparticles used in diagnostic.

### 2.1. Synthesis and Modification of Microgels and Hydrogel Microparticles

From the chemical point of view, hydrogels represent a very versatile class of materials. Indeed, material composition, functionalization and synthetic strategies can be easily adapted to the final purpose. In the context of hydrogels for sensing, the constituents can be classified as supportive monomers copolymerized with determinants of the interaction to have ultimately a multifunctional network. To this end, the base monomers lead the cross-linking and allow for the accommodation of other monomers that confer the sensitivity or the specificity toward a chosen target. For instance, highly hydrated long chain cross-linkers such as polyethylene glycols (PEG) became the base monomer of choice to build hydrogels with low-fouling properties enhancing the specificity of the capture elements thereof. PEG derivatives are extensively adopted for their good solubility in aqueous solution, low cost production at different molecular weights and chemical functionalities [26].

Here, we report the methods commonly used to synthetize microgels and hydrogel microparticles with particular attention to batch processing to obtain microgels while most advanced microfluidic tools will be described for the realization of lager microparticles.

#### 2.1.1. Batch Synthesis of Microgels

The main aim of microgel realization relies on the control of desirable size distribution, colloidal stability, and the presence of functional groups at specific locations (i.e., cross-linker, charged groups, or reactive centers) useful for further chemical derivatization [6,36,37]. The commonly adopted approach for microgel synthesis is (co)polymerization of vinyl monomers with cross-linker via radical polymerization. The polymerization procedures are usually radical photo or thermal-initiated by using appropriate initiators or for more complex architecture the synthesis can be performed via living controlled radical polymerizations (ATRP, RAFT etc.). Based on the particle formation mechanism, it is convenient to divide the diverse range of microgel preparation strategies into three classes: (i) homogeneous nucleation; (ii) emulsification; and (iii) complexation. Homogeneous nucleation refers to those preparations in which microgel particles are generated from initially homogeneous (or nearly so) solutions. Emulsification refers to those methods where aqueous droplets of a pregel solution are formed in an oil or brine phase and, in the second step, the droplets are polymerized and/or cross-linked into a microgel. Finally, microgels can be prepared by mixing two dilute, water-soluble polymers that form complexes in water.

**Figure 2:**
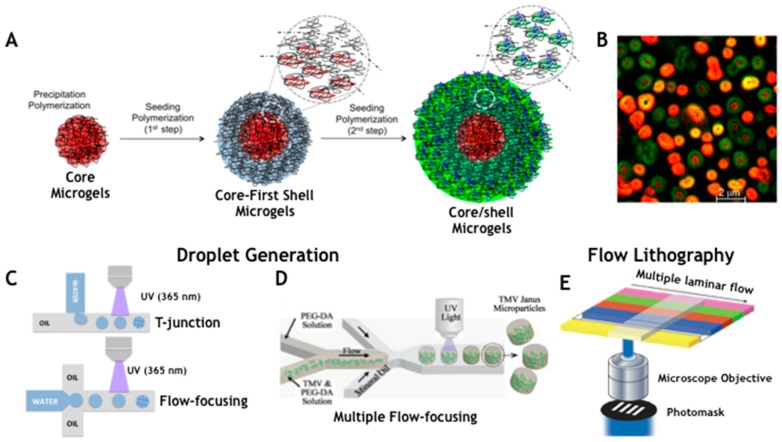

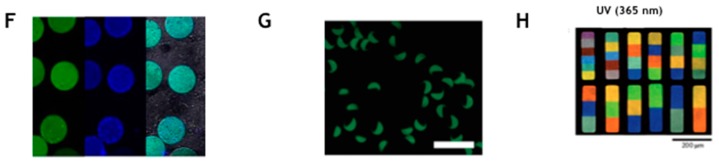
Microgel and Hydrogel Particle production by batch synthesis and microfluidics. (**A**) Core/shell microgel synthesis by multistep combination of precipitation and seed polymerization: the formation mechanism of microgels from the aggregation of precursor nanoparticles to the realization of (**B**) a spectral encoding system on microgels by copolymerization in different compartments of two different dyes. Emulsification in microfluidics through Droplet Generation systems: (**C**) simple T-junction and flow-focusing; (**D**) combination of multiple flow-focusing to obtain poly(ethylene glycol) diacrylate (PEGDA)-based TMV Janus microparticles; (**E**) Continuous flow lithography for the realization complex shaped hydrogel microparticles; (**F**) Hydrogel microparticles obtained by droplet generation; (G) encoding by Janus PEGDA hydrogels microparticles; and (**H**) graphical and spectral encoding combination on PEGDA microparticles obtained by flow lithography approach. (**A**) reprinted with permission from [36], copyright 2016 Wiley; (**B**) adapted with permission from [38], copyright 2015 American Chemical Society; (**F**) adapted with permission from [39], copyright 2016 Elsevier B.V.; (**D**,**G**) adapted with permission from [40], copyright 2010 American Chemical Society; and (**E**,**H**) adapted with permission from [41], copyright 2014 Springer Nature.

Particularly interesting are homogeneous nucleations where a solution of soluble monomer, including some type of cross-linking agent, is fed into the reactor and microgel particles form over the course of polymerization. A key requirement for discrete microgel particle formation is that the polymer formed must be insoluble in the solvent of polymerization. The mechanism of microgel formation under homogeneous conditions provided a radical (as a peroxosulfate species, i.e., ammonium persulfate (APS) or potassium persulfate (KPS)) is generated in solution to initiate the polymerization of monomer and cross-linker (Figure 2A). However, the insolubility of the oligomer network under polymerization conditions causes the growing polymer chain to phase separate, forming precursor particles that are not colloidally stable. As the aggregated precursor particles coalesce, the charged chain species tend to locate at particle–water interface. Therefore, as the aggregates grow, the surface charge density increases until a point is reached where the growing particle is colloidally stable with respect to similar sized or larger particles (primary particles). To achieve a monodisperse product, the primary particles must be formed at low monomer conversion. In later stages of polymerization, all newly formed precursor particles deposit onto existing stable microgels contributing to particle growth. There are few variables in the above microgel synthesis in this condition, thus it is difficult to obtain a wide range of average microgel diameters. Using a surfactant, it is possible to influence microgel particle nucleation and thus the final size. The role of surfactant is to stabilize the primary particles so that they are smaller than those prepared without. One-pot copolymerization allows obtaining microgels with non-uniform distribution of polymer chains with denser core and hairy surface ends. In addition, microgels with “core/shell” topology were also prepared via two-step “seed–feed” polymerization (Figure 2A). Virtually all chemical functionalities can be added to microgel particles by one-pot copolymerization, two-step “seed–feed” polymerization, and post-functionalization. The core/shell architecture resulted very interesting having hydrogel compartmentalization with differentiated proprieties in the core and the shell [24]. Two-stage polymerization allows obtaining microgels with a control over the radial distribution of the functional groups in the particle by adding polymer shell of the same or different structure or chemistry onto preformed core particles. Core/shell microgels realized in this way can exhibit very interesting features [35]. Indeed, multiple phase transition behavior has been realized since the shell can be synthesized using different co-monomers than the core. Further the dependence on the cross-linking density of the shell a gradient compression or “shrink-wrapping” of the core can be observed. Battista et al. [36] obtained core/shell microgels through a multistep procedure combining free-radical precipitation polymerization and seeding polymerization, using PEGDMA as main cross-linker. Microgels were designed with unique encoding system to match the requirements of a multiplex analysis. Two fluorescent dyes, with non-overlapping excitation and emission are incorporated together with anchoring groups on the outmost shell for subsequent conjugations, in this case carboxyl groups. The first step comprised a typical precipitation polymerization of microgels by copolymerization of cross-linker and fluorescent dye as methacrylate derivatives. Homogeneous nucleation is the mechanism of microgels formation: the decomposition of initiator produces free radicals that sustain the growth of polymer chains (Figure 2A). Upon the reaching a critical length, precursor particles form and grow until colloidal stability of microgels is reached. Monodisperse microgels (R1-core) are obtained (549 ± 7 nm) and subsequently used as seeds for synthesis of complex multishell microgels architectures. Thus, the synthesis steps were likely repeated by using R1-core as seeds to grow different additional shells. By this method, core/shell particles are obtained with no increase in polydispersity. All oligomers formed in solution adsorb on pre-formed core particles and through a mechanism somewhat similar to that for the core microgels the additional shells are formed. In this way, a first and a second shell of polyethylene glycol dimethacrylate (PEGDMA) copolymerized with different amounts of fluorescent methacrylate dye (Fluorescein *O*-methacrylate (Fluo)) and Acrylic Acid (AAc) were added to the R1 cores obtaining increasing size particles with low polydispersity (Figure 2B). Lyon group also proved the feasibility of this method to obtain hollow hydrogel capsules [30]. To this aim a degradable cross-linker, containing vicinal diols, was used to synthesize a core while the shell was growth with a conventional cross-linker. To accomplish this, the core is fabricated with a degradable cross-linker and the shell with a non-degradable one. The degradable cross-linker that have been used, which can be degraded by stoichiometric addition of periodate. After core degradation by periodate solution microgel showed a hollow structure as confirmed by dynamic light scattering (DLS) and fluorescence.

#### 2.1.2. High Throughput Production of Hydrogel Microparticles by Microfluidics 

Hydrogel microparticles for sensing purposes should be synthesized with finely controlled size, shape and chemical composition trying to match the requirements of liquid bioassays for the encoding, the capturing system and mass transport. Conventional syntheses of hydrogel microparticles are represented by dispersion, precipitation and emulsion polymerization procedures [42,43]. Such methods have intrinsic limits due to biomolecule incompatibility toward organic solvents and high temperature conditions needed. In addition the shape of such particles is restricted to spheres with even surface properties and often with a low monodispersity.

Recently, with the advance of microfabrication methods, hydrogel microparticles can be produced with tuned size and bulk chemical and physical properties by the use of miniaturized devices such as microfluidics. This approach represents the most promising methods for the production and the functionalization of monodisperse particles saving time, at low cost and with higher reproducibility [44]. Here, we report two methods to obtain hydrogel microparticles with complex composition and shape and potentially enabling high throughput production: droplet microfluidics and flow lithography (Figure 2C–E).

Microfluidic assisted methods are usually based on formation of a stable emulsion of an oil phase as continuous phase and a water phase constituted by preformed mix of monomers and biomolecules in miniaturized devices made in glass or polydimethylsiloxane (PDMS) [42]. For the sake of materials, high flexibility has been shown with mixture of vinyl monomers as well as natural polymers. Synthetic polymer networks obtained by cross-linking acrylate and methacrylate monomers have been widely used in particular starting from the use of PEG, like in the case of microgels, due to their inherent advantageous properties (solubility, anti-fouling, and large size range) assuring better control over the final composition of the micro-objects. The cross-linking of such monomers is performed by shining UV/Vis light on the flowing liquids in-situ or in specialized compartments activating a suitable photoinitiator based on hydroxyalkylphenone species (i.e., Darocur, Irgacure). A benzoyl free radical is produced upon the homolitic scission of C–C bonds in the photosensitive molecules that allows the cross-links formation in the gel. The intensity of light, the exposure time and the concentration of photoinitiators are determinant on the double bond conversion and in turn on the mechanical rigidity and the pores of the gels [45].

Microfluidic techniques can be divided in droplet generation based methods and flow lithography. Droplet forming micro-devices have been described since 2005 as continuous on-chip production of water-in-oil emulsions based on the breakoff of droplets in two-phases at T-junction or in flow-focusing geometries [46,47] (Figure 2C). The formation of droplet is the result of different variables such as flow rates, viscosity of the fluid, dimensions of the geometry, capillary number (Ca) and surface tension. The optimal design of a droplet generator for monodisperse and stable emulsion must have the control on each parameter that can be well predicted by simulations [39]. Capillary number defines the droplet formation process as the competition between the viscous stress and the surface tension of two immiscible phases. Such parameter defines critical values of (Ca) as Ca = μU/γ, where μ is the viscosity of the carrier fluid and γ the interfacial tension between the two immiscible phases. Choi et al. [48] found a limited range of Ca (0.5 × 10^−2^–0.5 × 10^−1^) and a flow rate of disperse phase (0.2–1.1 μL/min) where stable and monodisperse emulsions can be generated. Celetti et al. [39] performed simulation in a flow-focusing droplet generator showing how the change of each parameter of Ca affects the size of particles and the flow configurations after the junction. At high flow rates (*Q*_p_ > 8 × 10^−2^ uL/min) of the poly(ethylene glycol) diacrylate (PEGDA) phase the W/O emulsion could not separate uniformly from the junction due to the unstable hydrodynamic pressure and an elongation flow pattern is readily visible. When the flow rate is between 3 × 10^−2^ μL/min and 8 × 10^−2^ μL/min, the range values of Ca is equal to 0.005–0.055 and the aqueous falls in a regime of stable emulsions. Such geometries and configurations are often restricted to spheres, deformed spheres (rods, ellipsoids or discs) [49,50,51] or cylinders [52] due to surface-tension effects. Indeed the combination of multiple T-junctions or flow-focusing geometry allows performing double emulsion widening the option of integrating encoding and functionalities (Figure 2D,G). Droplet microfluidics facilitate large production of microparticles with a rate greater than 10^5^ particles per hours and a narrow size monodispersity less than 5–6% not possible with conventional methods, even though limited by the need of chemical mixtures of immiscible fluids and surface-compatible with microfluidic devices. Doyle group reported an innovative method for the continuous production of hydrogel particles under flow in a PDMS microfluidic device affording a throughput of near 18,000 particles per second and obtaining a high number codes by combining the graphical and spectral encoding [3,53] (Figure 2E). This approach has been defined as continuous flow lithography where a rapid and continuous production of hydrogel microparticles with unmet encoding schemes and multiplexing capabilities takes place from laminar nature of the microfluidic flow. The methods rely on rectangular microfluidic channel where multiple co-flowing streams pass over a pulsing UV light is projected through a mask from the objective to give complex shapes. Multiple shape and sizes have been obtained as well as differential chemistries are possible such as Janus particles. An evolution of such technology is represented by stop-flow lithography where the pumping system is actuated so that the flow is stopped for few milliseconds for the polymerization through a mask. In such a way Doyle group obtained particles with graphical and spectral encoding as well as with multiple capture probes positioned in different regions [41] (Figure 2H). These evolved methods have limitation in the polymerization step without deformation, the viscosities of liquids and the need for consistent flow, or adherence to channel walls [45].

### 2.2. Chemical Modifications

The functionalization of hydrogels is needed to allow the specific capture of a given target. In certain cases, the desired functionality can be directly added in the prepolymer mixture and allowed to co-polymerize. The feasibility of such approach depends exclusively on the stability of the chosen biomolecules or on the cross-reactivity of certain chemical groups present to the condition of polymerization. However, a small amount of co-monomers with carboxyl or amine functionalities are used during the polymerization step to allow further functionalization of hydrogels in a post synthesis phase. This approach is followed for conjugation of molecules that cannot withstand polymerization reaction conditions [36]. Most of the standard techniques for coupling small molecules, peptides, oligonucleotides, and proteins are applicable to microgels [53]. Indeed, microgels offer important advantages: first, microgels can be centrifuged and readily redispersed, which facilitates cleaning [39,54,55]; second, subtle changes can be followed by dynamic light scattering, which is sensitive to swelling, micro electrophoresis, and to surface charge; and, third, microgels are generally more colloidally stable than latexes and other nanosized support particles. The usual starting points for microgel derivatization are carboxyl or amine groups, but other functionalities have also been explored. Epoxy linkers can be been introduced during the polymerization by using a suitable monomer while aldehydes or thiols groups can be introduced after post polymerization procedure in aqueous mild conditions. More recently, click chemistry provided new tools for the functionalization with exceptional yields. Furthermore, click reactions are orthogonal with respect other approaches so that increasing the possibility of conjugating multiple molecules on one particle without side reactions. Thus, biotin, streptavidin, proteins, and oligonucleotides have been conjugated to microgels [26,36].

### 2.3. Hydrogel Characteristics for Biosensing 

**Figure 3:**
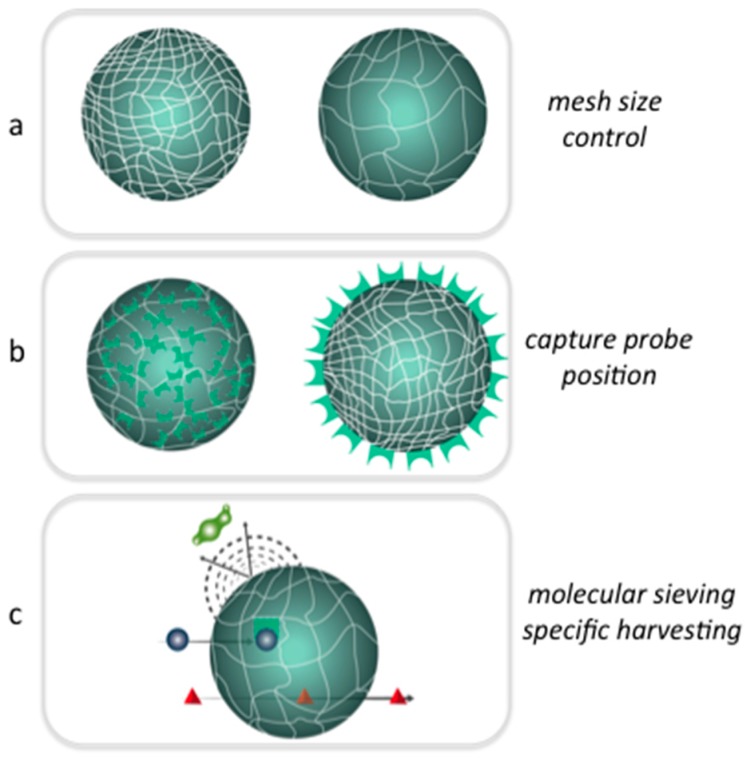
Hydrogels can be tuned in their physical and chemical properties to perform specific functions matching the need of the final applications: (**a**) tunable control of mesh size from polymerization reaction conditions; (**b**) versatile chemistry to allow the conjugation of capture molecule in different position; and (**c**) molecular sieving coupled to a specific capture of a given target obtained by changing the mesh size and cross-linking a ligand inside the hydrogel matrix.

In this section, we report on the chemical and physical properties of hydrogels that affect primarily their behavior in biosensing applications. In fact, the three-dimensional, and eventually responsive, nature of the polymer networks plays a key role in bead-based assays and when considering hydrogel materials in biosensing applications a number of concerns have been to take into account. Particularly important are the issues related to the capability of capturing the given target and to the mass transport inside the network closely connected to the swelling behavior, the probe density and diffusion of solutes (Figure 3).

The performances of a hydrogel in sensing, as well as in other context, are dependent to a large extent on its bulk structure and on the interaction with solvent molecules [19]. The knowledge and the control at molecular level of hydrogel structure is then of crucial importance in engineering a well-defined network that allow for the transport of selected target for example based on the size and the chemical content. Hydrogels can work as molecular sieving representing a physical barrier to the entrance of large molecules or allowing the free motion to the small molecule and repelling unspecific binding (Figure 3c). The specific binding can be realized thanks to the insertion during the polymerization or in a post modification step of molecular catcher inside the bulk or on the surface. Furthermore, it is possible to implement mechanisms of signal transduction upon a specific interaction based on transition between a swollen and collapsed state.

#### 2.3.1. Swelling Behavior and Porosity

The control of the hydrogel structure at molecular level determines how a molecule moves inside the matrix and how can be implemented active mechanisms for the capture and detection of particular targets. Primarily, the access of target molecules to the immobilized ligands has to be assured while maintaining a structural integrity of the hydrogel matrix. This prerequisite defines the need to optimize the monomer composition mix, the porosity, the particle rigidity and the swelling behavior. In general, the swelling behavior and the porosity of hydrogels can be easily tuned according to the starting material properties (i.e., monomer concentration, cross-linker mass weight) and it is also possible to implement the dynamic change of the porosity in response to pH, ionic strength or temperature. However, in engineering hydrogel molecular structure for biodetection applications, polymers, that are responsive to environment stimuli other than water content, are avoided in order to preserve porosity, chemical background, and, therefore, target diffusion. Usually for the purpose of diagnostic assays, hydrogels are realized to accommodate a capture molecule inside a non-fouling network with a predetermined mesh size with a cut-off for certain molecules known as interferents. The characterization of the network structure of a hydrogel passes through the determination of the polymer fraction (v_2_,s), the molecular weight of the polymer chain between two neighboring cross-linking points (Mc) and the correlation distance between them, also known as mesh size (ξ) [36,56]. The nature and the amount of polymer fraction defines how a hydrogel is capable to imbibe and retain water, while the Mc and ξ are averaged parameters that defines the accessibility and the transport of molecular species in the network. However, all these parameters are described theoretically by the equilibrium swelling theory and the rubber elasticity, while a variety of techniques are available to experimentally measure and verify them [57,58].

For bulk hydrogels, the structural characterization is usually carried out measuring the degree of hydration (swelling measurements) by weighting the amount of water uptaken by the polymer and converting it in the volumetric fraction according to the so-called equilibrium swelling theory developed by Flory and Rehner and then modified by Peppas and Merrill [59]. However, this approach is not straightforward for microgels, as inherent difficulties in the canonical measurements of swelling of such microparticles [60]. Recently, Battista et al. demonstrated the direct determination of swelling on core/shell microgels through atomic force microscopy (AFM): Mc and ξ were determined from the topographic imaging measuring the actual volume by “Laplacian volume” occupied by the single microgel [36]. Once volumes were determined, swelling ratio (Q) was obtained for both swollen (Q_s_) and relaxed state (Q_r_):

(1) $$Q_{s}=\frac{V_{s}}{V_{d}}{\;Q}_{r}=\frac{V_{r}}{V_{d}}$$

where V_d_ is the dry volume, V_s_ the volume in the swollen state and V_r_ in the relaxed state. These data can be considered as the starting input for the equation to calculate the molecular weight between cross-links in presence of water according the following formula [61]

(2) $$\frac{1}{{\bar{M}}_{c}}=\frac{2}{{\bar{M}}_{n}}-\frac{\left(\frac{\bar{v}}{V_{1}}\right)\left[\ln\left(1-v_{2,s}\right)+v_{2,s}+\chi_{1}v_{2,s}^{2}\right]}{v_{2,r}\left[{\left(\frac{v_{2,s}}{v_{2,r}}\right)}^{1/3}-\left(\frac{v_{2,s}}{2v_{2,r}}\right)\right]}$$

where M_n_ is the number average molecular weight of the un-cross-linked polymer, χ is the Flory polymer-swelling agent thermodynamic interaction parameter (for PEG/water is χEG/water), v is the specific volume of the polymer, and v_1_ is the molar volume of water [62]. v_2,s_ and v_2,r_ represent, respectively, the polymer volume fraction in the swollen and relaxed state. The latter is defined as the state of the polymer immediately after cross-linking, but before swelling [61,62] and are mathematically the inverse of swelling ratio (Q), calculated as follows:

(3) $$v_{2,r}=\frac{V_{d}}{V_{r}}\;\mathrm{and}\;v_{2,s}=\frac{V_{d}}{V_{s}}$$

Once M_c_ was calculated by Peppas and Merril equation, the mesh size can be derived:

(4) $$\xi =v_{2,s}^{-1/3}{\left({\bar{r}}_{0}^{2}\right)}^{1/2}$$

where ${\bar{r}}_{0}^{2}$ is the value of the end-to-end distance of PEG chains in the unperturbed state and it is calculated through the Flory characteristic ratio, or rigidity factor, C_n_ (C_n_ = 4) [63]:

(5) $$C_{n}=\frac{{\bar{r}}_{0}^{2}}{{\mathrm{Nl}}^{2}}$$

where N is the number of links of the chains, given by:

(6) $$N=\frac{2{\bar{M}}_{c}}{M_{r}}$$

where M_r_ is the molecular weight of the repeating unit of the polymer (M_r_ = 44) and l is the C–C bond length (l = 0.146 Å) [63,64].

#### 2.3.2. Probe Density

The increased surface as well as the three-dimensional molecular structure of the hydrogel allows for a versatile and flexible possibility for the inclusion of probes for the target capture.

Polymeric hydrogel network permits a higher capability to conjugate probes with respect to other approaches such as surface deposition, starting from the same spotting solutions. Hydrogel microchips endowed by fluorescent antibodies, at the same solution concentration, showed higher emission than the surface protein microchips related to an immobilization density as high as 10**^−^**^4^–10**^−^**^3^ M, never attainable for polymer surfaces. Moreover, the mean distances between immobilized probes can be assessed as d_gel_ = [p]^−1/3^, while d_surf_ = [p]^−1/2^, where p indicates the probe concentration [65].

Assuming a target–probe reaction following first-order Langmuir kinetics, a higher concentration of probe bring to a greater number of target capture at equilibrium with a resulting better sensitivity. The restrictions related to the binding kinetics rates impose the condition [p]Ka < 10^3^–10^4^ and partially restrict the maximal capacity of gel [65,66].

As an example, in the case of large molecules (i.e., antibodies), the upper limit of probe immobilized on a surface attains to a density of (3–4) × 10^12^ molecules/cm^2^, whereas in the case of hydrogels concentrations of 10**^−^**^4^–10**^−^**^3^ M in the vertical projection and an effective surface densities of about 10^14^–10^15^ molecules/cm^2^ considering a hemisphere with the radius 75 um.

In the case of polymer synthesis, the upper limit concentration is mainly dictated by processing parameters such as probe solubility with concentration up to 300 um in PEGDA systems [26]. Furthermore, finite probe incorporation efficiency has been reported during radical polymerization reactions with yields down to even 10%. In such conditions the probe consummation is higher than in microarray technology, but depending on particular particle array systems. In particular, in the case of PEG microgels 7 pmol/assay is needed versus 50 pmol/assay in the case of Luminex^®^ system and about 1 pmol/assay in traditional microarray [67,68].

#### 2.3.3. Target Diffusion in Hydrogel

Particular attention must be attained in the evaluation of the diffusion of biomolecule targets in hydrogel matrices. Such evaluation contributes to the investigation of structural parameters of the hydrogel with respect to the trafficking biomolecules as well as to provide necessary coefficients to compute the mass transport within the gel during a typical assay [69].

In free solution and in absence of any interactions with other target biomolecules, the diffusion process is governed by Stokes-Einstein relation:

(7) $$D_{0}=k_{B}T/\left(6{\pi \eta R}_{h}\right)$$

where k_B_ is the Boltzmann constant, T is the temperature in kelvin, η is the solvent viscosity and R_h_ is the hydrodynamic radius of the target. In hydrogels, the diffusion is explained by different models, related to the nature of the biomolecule target.

In the case of oligonucleotides considered as Gaussian chain for Rg < a/2 (Rg stands for gyration radius), the diffusion coefficient is described by Zimm model [70], where the macromolecule migrates in ellipsoidal conformation, Rg is the gyrations radius of such biomolecules, can be calculated by chemical characteristics of the chain [71], and a is the mean gel pore size according to polymer theory:

(8) $$D_{0}=0.196{\;k}_{b}T/\left({\eta R}_{h}\right)\;\approx {\;N}_{0}^{-1/2}$$

If Rg > a/2, the reptation theory describes the movement of an unattached chain by Brownian motion in a multi-chain system forming the gel. The lateral movement of the chain is limited by gel fibers with a resulting loss of entropy that brings the chain to migrate inside a tube with length L = Na, where N is the number of pore occupied by the chain. In such scenario:

(9) $$D=k_{B}{T\;a}^{2}/\left(3{{\;N}_{k}}^{2}\zeta_{k}{\;b}^{2}\right)\;\approx {\;N}_{0}^{-1/2}$$

a represents the pore size, N_K_ is the the number of Kuhn segments, ζ_K_ is the friction coefficient of a Kuhn segments, and b is the Kuhn length.

For proteins considered as rigid beads, the stochastic model proposed by Ongston et al. [72] address the diffusion of spherical particles in a fibers array with:

(10) $$D/D_{0}=\exp(-\varphi^{\frac{1}{2}}{\;R}_{h}/r_{f})$$

where D_0_ is the diffusion coefficient in solution, r_f_ the fiber radius and ϕ is the volume fraction of the fibers.

#### 2.3.4. Mass Transport Equations

To properly set-up a bioassay occurring in a hydrogel, it is crucial to investigate mass transport and consider relative equations.

If we assume a target (T) diffusing into a hydrogel particles provided with probe (P) to form a complex target TP. D is the coefficient diffusion of the target in the hydrogel matrix as previously introduced. V_s_ is the sample volume in contact with the hydrogel of volume V. The dissociation constant of the complex TP is K_D_ = k_d_/k_a_ where k_d_ and k_a_ are respectively the first order dissociation and association constant for the given complex [71]. If the sample is well mixed the target concentration in solution is only dependent with time. If V_s_ is order of magnitude higher than V, the target concentration in solution can be considered constant and unaffected by complex formation.

Therefore, equations governing the species concentration are the following:

(11) $$\frac{\partial_{\left[T\right]}}{\partial_{t}}=D\nabla^{2}\left[T\right]-k_{a}\left[P\right]\left[T\right]+k_{d}$$

(12) $$\frac{\partial_{\left[P\right]}}{\partial_{t}}=-k_{a}\left[P\right]\left[T\right]+k_{d}\;\left[\mathrm{PT}\right]$$

[PT] = [P]_0_ − [P] (13)

(14) $$\frac{\partial \left[T\right]}{\partial t}\mathrm{Vs}=-\iint^{\text{​}}D\nabla \left[T\right]n\;\mathrm{ds}$$

where n is the unit versor normal to hydrogel surface. Usually, at t = 0, the target is completely in the sample solution, while the probe is uniformly distributed in the hydrogel matrix. The boundary conditions come from geometry and partition coefficient of the specific system.

Scaling considerations can be considered to simplify the complexity of the nonlinear and couples equations. Dimensionless groups can describe the rate of association versus the diffusion in term of *Damkohler* number Da = k_a_[P]_0_/(D/L^2^), where l is the characteristic length for the diffusion path, the ratio of the target to probe molecules g = [T]_s_V_s_/[P]_0_V and the relative strength of complexation k = K_D_/[T_s_]_0_.

In the case of short oligonucleotide k_a_ (10^6^ M^−1^s^−1^) is much higher than D (10^−11^ m^2^/s), therefore Da >> 1 if [P]_0_ is about 10^−6^ M and L around 10^−6^ m; even at low amount (<10^−18^ mol) the probe can be considered in large excess, therefore g << 1, while hybridization is very strong at initial target concentration (k << 1) if considering K_D_ in the order of 10^−13^ M and [T_s_]_0_ around 10^−11^ M [71].

In the case of larger oligonucleotide, k_a_ is around 10^3^ M^−1^s^−1^, while assuming that D is the same of other biomolecules with the same Rg or R_H_ (10^−8^–10^−10^ m^2^/s) [73], Da can be in the unity range if [*P*]_0_ is about 10^−6^ M and L around 10^−6^ m; the probe can be considered in large excess even at low amount, therefore g << 1.

In the case of proteins, antibodies or peptide probe for target capture can be immobilized in the gel in excess. k_a_ is in the range of 10^4^–10^9^ M^−1^s^−1^, in the case of protein–antibody complex with D in the range of 10^−8^–10^−10^ m^2^/s, while k_a_ is in the range of 10^3^–10^5^ M^−1^s^−1^ in the case of protein-peptide complex with *D* in same range [74,75]. Therefore, if [*P*]_0_ is about 10^−6^ M and L around 10^−6^ m Da can changes attaining also values lower than 1. K_D_ is in the range of 10^−8^–10^−10^ M in the case of protein–antibody complex, while 10^−6^–10^−7^ M in the case of protein-peptide so that the relative strength of complexation can also largely vary.

When Da >> 1, the penetration distance for target into hydrogel can be approximated to L/Da^1/2^, that could be much smaller that characteristic size of the hydrogel. This determines a predominant mass transport confined to the surface, creating an uneven distribution of target in the hydrogel volume V, with subsequent limitation of the diffusion towards the inner part of the volume.

## 3. Microgels and Hydrogel Microparticles in Multiplex Assays

The use of microparticles revolutionized the existing methodologies in term of time and cost, sample amount and sensitivity. Microarrays and ELISAs mainly benefited from the microparticles bringing to the market well assessed technologies increasingly easier to perform. Hydrogels microparticles and microgels are continuously evolving the concepts of the liquid assays representing a material platform with enhanced capability to overcome specific needs (for a detailed comparison on the performances and limit of detection (LOD) see [26]). Here, we mention some key elements where the hydrogels outperform over other material in realizing multiplex assays.

One of the major concerns in multiplex analysis is the encoding. Indeed, in the case of 2D arrays, the identity of each probe is determined by positional encoding, where the location on the grid univocally identifies the target [4].

The great advantage of using microspheres relies on the flexibility of manipulation in three dimensions, but this precludes the positional encoding. Therefore, each microparticle is endowed by a unique code that defines the capture molecules conjugate on the surfaces. The encoding must address a number of requirements: it must be optically read; inert and robust at the operational conditions; robust, with low error rate; capable to produce a large number of unique combinations; implementable on materials of few micron in size and suitable for low-cost mass production. Several technologies have been adopted for encoding beads, here we present few example of optical and graphical encoding that can be easily implemented in microgels and hydrogels [76].

Most of the bead-based assays commercially available are comprised of polymer microspheres with one or more fluorescent dyes physically entrapped. For the sake of multiplex assays a reporter dye is required to emit in a region of the spectrum free from the emission of encoding. However, by dye entrapment of distinct emission spectra at different ratios, spectral encoding allows recognizing univocally hydrogels. Multiple dyes at different concentrations enable a number of unique codes according to X^N^–1 (X^N^ if a zero level of all dyes is included), where X is the number of dyes used and N the number of available concentration levels of each individual dye. Different examples provide microgels and hydrogels where fluorescent dyes are incorporated during the polymerization as acrylic derivatives. Limitations in that approach rely on the applicability to more efficient fluorophore and the conditions of synthesis of hydrogels as the radicals formed during the polymerization affect their emission. More stable fluorescent emission have been shown by semiconductor nanoparticles, also known as Quantum Dots (QD) [77]. The emission line width of quantum dot is usually narrower with respect organic dyes, and the peak position of the emission can be finely tuned by varying the radius of the quantum dot, enabling high number of unique encoding. On the contrary, the excitation spectrum of a QD is very broad [78], so that simpler the reading optics have to be realize by using a single wavelength to excite different sized QDs. As an example, by using six different emitting QDs at six ratios produces 6^6^ − 1 = 46,655 unique combinations. [79]. One of the major drawbacks of the encoding through fluorescent dye/QDs is mainly related on the reliability of the emission reading that could appear different on the same population of beads. Thus, such methods can be subjected to variability on fluorescent compounds in the beads causing variations in the fluorescence intensity. Further the interference between many emission wavelengths of fluorescent dyes used as target binding and encoding reporters may impair a correct detection and the number of codes. The encoding capacity of these techniques is therefore limited to a reasonably small number of codes.

As for the graphical encoding, hydrogel microparticles have been microstructured with patterns constituting a code. Pregibon et al. [3] demonstrated the encoding, analyte attachment (to the particles) and reading of the codes all within a microfluidic system. The stop-low lithography approach is able to produce particles with physical patterns and fluorescent dyes. In that way multiwavelenght/spatial fluorescent encoding expands the number of possible combinations. Indeed considering for a barcode with N spatial position elements and C colors, is given by (C^N^ + C^N^/2)/2 when N is even and (C^N^ + C^[N+1]^/2)/2 when N is odd. Accordingly, combining four dye colors on six positions, the number of possible unique codes (non degenerate code attainable) is (4^6^ + 4^3^)/2 = 2080 [41]. This number corresponds to all possible color combinations, considering that symmetrical barcodes can be read from both directions, as there are no orientation markers for reading.

### 3.1. Liquid ELISAs : Antibody Based Detction

In the biomarker analysis field the need for multiplex analysis has pushed the researchers to adapt classic design of immunoassays for protein or antibody detection onto hydrogels. The exploitation of hydrogels as supportive materials evolved the concept of liquid-ELISA [53]. Appleyard et al. realized for the first a sandwich immunoassay for multiplexed detection of a panel of three cytokines (IL-2, IL-4, TNFα) on barcoded PEGDA particles. Encoded particle for each target were provide with the correct antibodies and mixed to the sample [53]. The reporting system was realized through a secondary biotinylated reporter antibody at sandwich with the captured target and showed with a streptavidin–phycoerythrin conjugate. The capture was realized directly on spiked samples in fetal bovine serum. The background of the signal was very low even in a complicated matrix, that the use of hydrogel avoided the need for prior purification of the sample. A comparison of such assay with commercial planar assays and Luminex bead-based assay proved more advantageous. First the similar limits of detection in the range of pg/mL were attained but without enzymatic signal amplification and requiring a lower number of beads to have a statistical significance. Further, a direct capture of antibodies was realized by shape-encoded hydrogel microparticles of PEG functionalized with IgG and IgM antibodies. In this case, the duplex assay showed high selectivity in detecting the chosen targets with a good linear correlation at concentrations up to 500 ng/mL. In the same way, Yang et al. realized a non-competitive immunoassay on encoded silica–hydrogel hybrid beads for the detection of tumor markers [80]. The fluorescence of encoded beads with labeled antibodies were measured before and after, compared to the levels in solution, and reported to a calibration curve for each marker.

More recently, Choi et al. [81] reported on the multiplex detection of epigenetic alterations in gene expression by using QDs encoded hydrogel microparticles endowed by specific antibodies for mutated histones. The potential of such methodology was demonstrate on cocaine-exposed mice by recognition at same time of: (1) acetylation of lysine 9 of histone 3 (Ac-H3K9); (2) dimethylation of H3K9 (2Me-H3K9); and (3) trimethylation of H3K9 (3Me-H3K9) present in three distinct regions of the brain. Such epigenetic assay based on hydrogels allowed the relative quantification of variants histones from low volume sample (10 μL of each brain lysate) with a protein content around 1 μg/μL per mouse.

More recently, aptamers and peptides are at base of more innovative strategies for protein sensing on hydrogels. Cusano [8] et al. realized a microfluidic tool-box to integrate binding peptide sequences on core/shell magnetic PEGMA microgels for the capture of proteins in serum. In particular, the selection of a peptide by Phage Display toward the soluble protein factor rhTNFα was demonstrated successful and the incorporation in a highly hydrated environment provided by hydrogels of a 12-mer peptide (called G6) allowed the selective capture in complex medium. The fishing out of the specific biomarker proved highly selective in serum in a sandwich assay with FITC-antibody and enhanced by the matrix hydrogel matrix.

**Figure 4:**
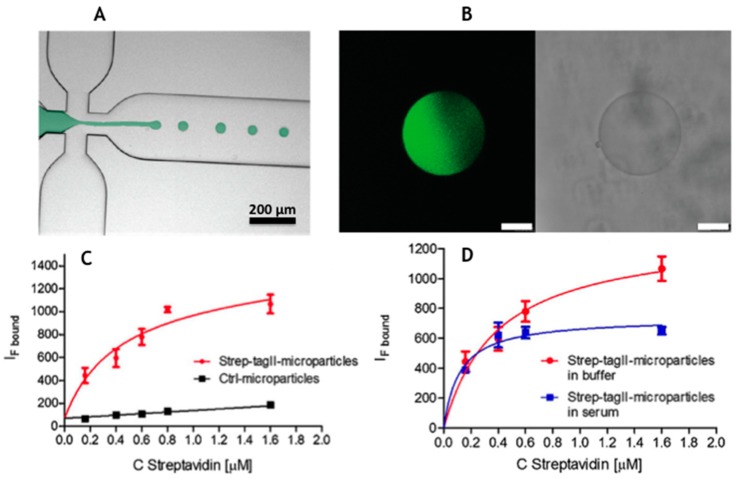
(**A**) Hydrogel microparticles with Strep-Tag II copolymerized with PEGDA are realized by flow-focusing droplet generation approach; (**B**) the generated droplet photopolymerized inside the microfluidic channel provide monodisperse particle (diameter 88 μm, sd 5 μm) with uniformly distributed peptide sequence co-polymerized inside the gel (size bar is 20 μm); and (**C** and **D**) in the lower part of the figure are reported the isotherm binding curve of the Atto-425 Streptavidin captured on by Strep Tag II microparticles over a control in PBS and in serum (adapted with permission from [39], copyright 2016 Elsevier B.V.).

Celetti et al. [39] demonstrated the easy and fast realization, in a single step, of functionalized monodisperse PEG microparticles with a peptide within polymeric network for protein capture (Figure 4). In particular, Strep-tagII peptide sequence was incorporated by droplet microfluidic in order to create a functional microparticles and the protein binding was shown dose-response and selective in serum with a K_d_ a slow as 0.12 uM. Such performance was ascribed to the capability of the polymeric network to offer antifouling properties, thus improving the specificity of the capture. As proteins are fragile molecules multiplexed immunoassays are usually more complex paving the way for poor reproducibility, cross-reactivity and non-specific binding issues so that the development of assays for nucleic acids results more straightforward [7].

### 3.2. Oligonucleotides: MicroRNA and ssDNA

MicroRNAs (miRNAs) are a class of short non-coding RNAs molecules with regulatory tasks on the genome expression [82]. The existence nucleic acids have known for 65 years as circulating biomarkers in plasma and serum has been, but only recently miRNAs have been observed body fluids and exploited as circulating biomarker for different disease. Circulating biomarkers in body fluids present advantages in the clinical diagnostic practice for the easy access as in the case of the prostate antigen (PSA, KLK3) is widely used to screen for prostate cancer while troponin (TNNT2) is a valid marker for myocardial infarction. However, the discovery of new biomarkers base on proteins is challenging as their low abundance in samples and problems to develop high affinity capture reagents. On the contrary, miRNAs proved advantageous over protein biomarkers in many of these regards.

More efforts have focused on miRNAs in cancer as they were first observed in cancer cell lines [83,84,85,86,87] and so far “liquid biopsy” was conceived as a way to make a complete profiling of a cancer disease from a drop of blood [88,89,90]. More recent evidence widens the applicability of miRNAs as biomarker also for other diseases [91,92,93].

As an example, in diabetes mellitus (DM), there is a need of robust biomarkers for monitoring cardiovascular diseases, kidney failure, and other closely related pathologies with high impact on diagnosis and prognosis of the primary disease. Different miRNAs have been correlated in the development of β-cell and in diabetes pathogenesis, or in insulin resistance in target tissues such as adipose, muscle, and liver cells [94,95].

In the same way, development and progression of cardiovascular diseases is accompanied by a plethora of complications and the “myomiRs” were individuated as a set of miRNAs with direct role in cardiovascular development, function, and disease [96,97,98]. The aforementioned pathologies better exemplify the potential in the identification of circulating miRNAs representing potent biomarkers useful not only in the diagnosis phase but also in monitoring the progression of the disease.

However, in all above mentioned cases the detection of oligonucleotides is usually performed by well-known technologies based on polymerase chain reaction (PCR, qRT-PCR) [99], oligonucleotide probes such as molecular beacons (MB) [99] and double strands (ds) [100]. In particular, qRT-PCR shows high sensitivity (down to 10 fM) and high-throughput ability at expense of time-consuming steps of extraction, amplification, and calibration, very often sources of biases that may compromise the assay accuracy [101]. A direct and absolute quantification of miRNAs in body fluids is particularly needed even though challenges are represented by their small size, high levels of sequence homology, complex secondary structures, demand of high sensitivity in a range between atto- (aM) to femtomolar (fM), specificity and multiplexing [9].

In this context, Chapin et al. realized an innovative universal labeling system for hydrogel microparticles adapted to the detection of microRNA [102]. Unlike proteins where sandwich assays have been implemented in hydrogels, nucleic acids do not present several epitopes to be targeted. The approach developed by Chapin makes use of miRNA probes comprised of two parts: one region specific for miRNA target and one for the universal label. In a two-stage assay format, hydrogel particles are incubated first with sample and then with the report label. In the case of hybridization of both molecules, an enzymatic ligation step firmly link the universal label to the bound miRNA target that finally allow the detection by labeling the biotin groups by a avidin fluorophore. This method claims to eliminate the bias of amplification step of PCR-based approaches as well as the pre-labeling phase of the target.

Similarly, an amplification scheme was set using the same approach by cell rolling circle amplification [103]. In this case miRNA targets bound to the probe are amplified based on the universal linker, rather than the target, limiting biases. MiRNA with a detection range of six orders of magnitude with a LOD at sub-femtomolar concentrations in complex matrices was reported.

**Figure 5:**
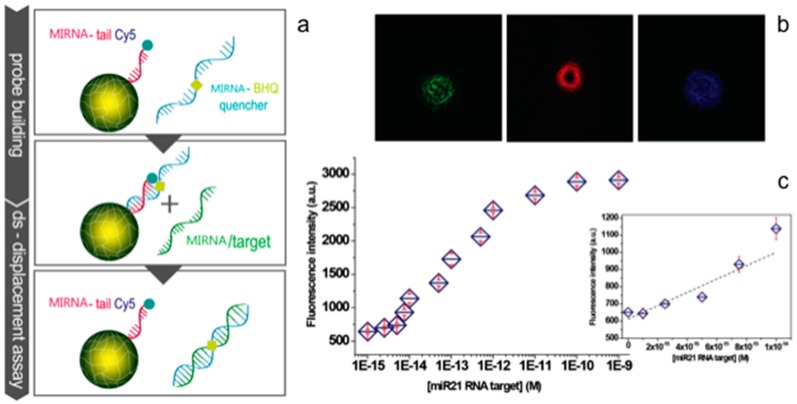
Microgel platform for microRNA detection based on a strande displacement mechanism: (**b**) the building of the probe on an encoded microgel; (**a**) the strand displacement upon contact with target microRNA and fluorescence recovery on the particles; and (**c**) the distinct fluorescent channels for fluorescein and rhodamine for the encoding and Cy5 for the detection of binding; in the lower part is reported the titration curve of the recovered fluorescence in response to increasing amounts of microRNA (the inset show the limit of detection (LOD) = 2.6 fM) (adapted with permission from [38]. Copyright 2015 American Chemical Society).

Moreover, unmet performances in miRNA detection have been achieved by coupling innovative probes on encoded core/shell microgels [38]. The use of the microgel platform corresponds to a 10^5^-fold enhancement in sensitivity (2.6 fM) compared to what is achieved by the same probes not conjugated to microgels (172 pM) because of the confinement of the fluorescence emission in a small volume (Figure 5).

The use of microgels leads to a simple and absolute quantification in serum extracts without target amplification steps, internal normalization, or primer optimization with a higher precision than qRT-PCR. The microgel performance is not significantly affected by the complexity of the serum. The microgel, indeed, provides a highly hydrated environment with antifouling properties producing an enhanced nucleic acid hybridization, in comparison to other solid surfaces [54,104].

**Figure 6:**
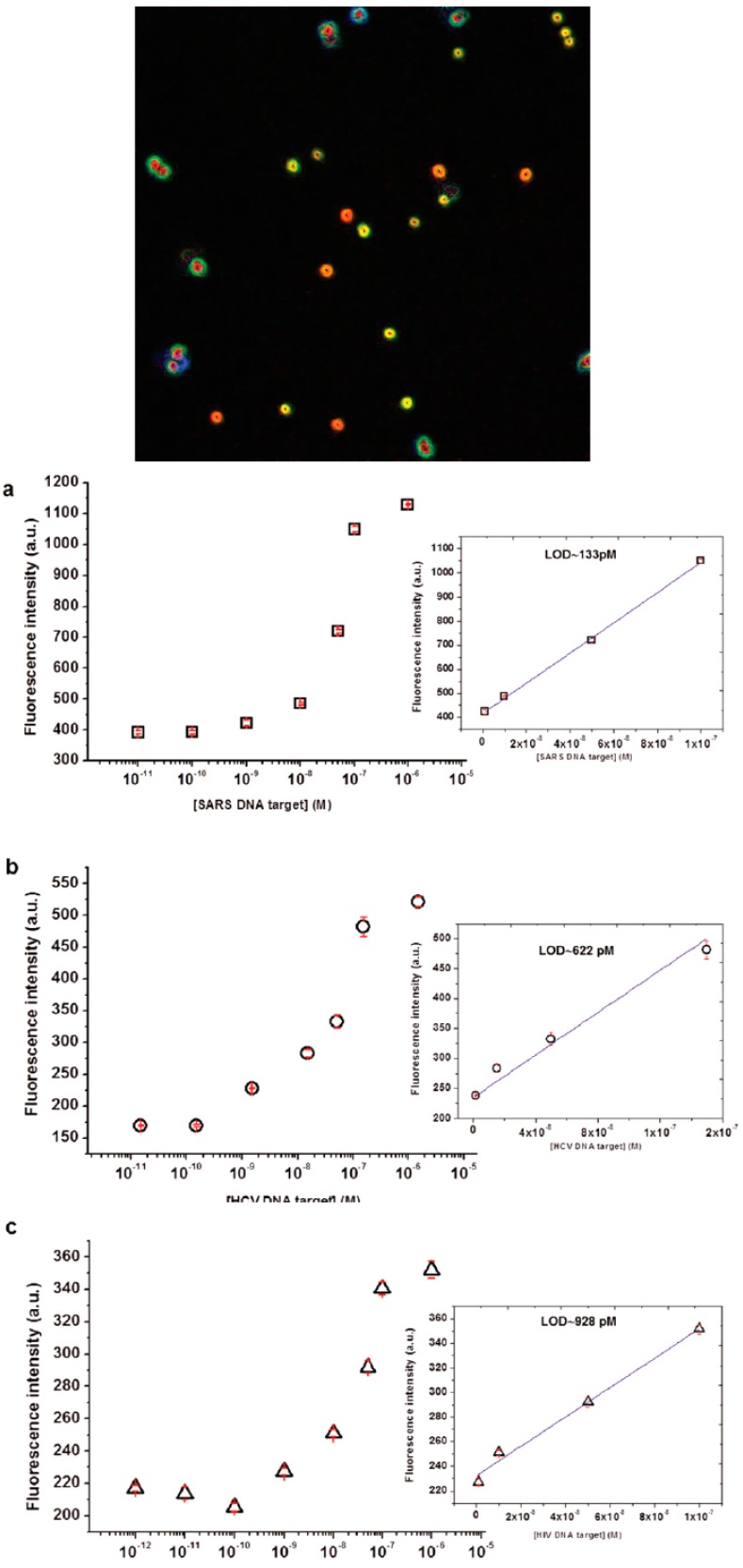
(**Upper**) Double encoded core/shell PEG microgels are imaged by fluorescence microscopy. Microgels are obtained by multistep synthesis allowing the realization of concentric shells with no spectral overlap between with the reporting system provided by suitable probe. (**Bottom**) The binding isotherms of ssDNA target of: (**a**) HIV; (**b**) SARS; and (**c**) HCV (Reproduced with permission from [55], copyright 2016 The Royal Society of Chemistry).

Another example of specific capture by oligonucleotide probes have been reported by Aliberti et al. [55] for the multiplex detection of HIV, SARS and HCV. In that case, a single strand DNA represents the target after a reverse transcription step of specific unique sequence of viral genome. The encoding system embedded on core/shell microgels of around 1 μm in size is realized through the compartmentalization of two dyes fluorescein and rhodamine copolymerized in a multistep batch precipitation and seed polymerization. Such approach can generate the minimum combination to perform a multiplex assay toward three different targets with a great sensitivity in a direct way, circumventing the amplification steps of the conventional PCR protocols. This system proved a limit of detection in the rage of 0.1–0.9 nM compatible with manifest progression of a viral infection (Figure 6).

According to the scheme of encoded core/shell microgels for oligonucleotide, Dannhauser et al [105] provided an easy tool for a quasi real-time fluorescence detection of single microgels in microfluidic flows using non-Newtonian fluids at a low concentration level is recently reported. In particular a cost-effective and biocompatible viscoelastic fluid was used to achieve optimal microgel alignment in the centerline of a straight channel. In such a way, a robust and simple readout system from up to nine microgel barcodes was assembled. As the detection of miRNAs can be easily tuned, the system can be exploited to other biomarkers provided the barcode structure. Moreover, the user can adjust the readout speed, according to the performance of any microscopic system, conferring high versatility to such approach. In particular, hundreds of encoded microgels were identified and counted in-flow and specific miRNA target quantified demonstrating the specificity of the assay in multiplex measurement conditions, with a detection of miR21 concentration down to pM.

## 4. Conclusions and Outlooks

In conclusion, hydrogels in the form of microparticles or colloidally stable submicron microgels proved effective in the detection of different kind of biomarker. Indeed, different sets of highly sensitive assays can be realized benefiting from the intrinsic properties of such materials. Hydrogels microparticles and microgels in general can be considered as a universal materials platform where is possible to realize a toolbox to tune chemical and structural properties. Thus the approach is versatile and flexible to allow the proper inclusion of probes for the target capture matching the increasing demand of commercial detection systems and devices. Therefore the advantageous properties of hydrogels are related to the three-dimensional molecular structure forming a liquid-like environment lowering the kinetic of interaction and maximizing the insertion of molecular catcher. Furthermore such materials represent molecular sieves with tunable mesh size to realize selective fishing of molecules based on their mass.

The use of such materials in multiplex bead based assays is considered mature for commercial product exploitation. The ever increasing demand of integrated, miniaturized and easy to use devices applications require materials with superior properties and versatility to be declined in different context. According to a bead-based assay, the need to encode, manipulate and record fluorescence images from hundreds of microgels or hydrogel microparticles is demanding and time consuming, especially in multiplex assay. Furthermore, the stability and time-dependence of probe–target complex bring to issues related to the reading conditions and assay time. However, thanks to the intrinsic nature of such materials, unmet performances have been achieved in terms of limit of detection and step assay allowing direct ultrasensitive detection in complex media for protein and oligonucleotides.

Current techniques allow converting the recognition event from single particles into electrical or optical signals for detection requiring sophisticated equipment and trained staff for analysis. In particular, despite the cost and ease of performing analysis on electrical platforms, the detection limit is poor. On the contrary, optical read-out offers improved sensitivity demanding for sophisticated operations, which restrict the applications for real-time online detection. In this regard, a trend towards non-Newtonian fluids, which allow viscoelastic 3D particle migration towards a centerline in straight or circular microchannels, proved a promising and viable way toward integrated devices and the ease of use [106,107,108,109,110]. In fact, viscoelastic induced particle alignment—at low volume concentrations—permits the optical observation of target particles in a simple and cost-effective way [111,112]. Although viscoelastic focusing presents similarities to cytofluorimetry, the particle centering through suitable colloids proved successful in particular with submicrometric microgels where cytofluorimetry can fail. However, such technology is promising to foster the development of miniaturized new devices for point-of-care widening the range of application not only in poor clinical settings.
